# ASCA and ANCA among Bedouin Arabs with inflammatory bowel disease, the frequency and phenotype correlation

**DOI:** 10.1186/s12876-018-0884-x

**Published:** 2018-10-20

**Authors:** Naim Abu-Freha, Wafi Badarna, Ina Sigal-Batikoff, Muhammad Abu Tailakh, Ohad Etzion, Jaber Elkrinawi, Arik Segal, Alex Mushkalo, Alex Fich

**Affiliations:** 1 The Institute of Gastroenterology and Hepatology, Soroka University Medical Center and the Faculty of Health Sciences, Ben-Gurion University of the Negev, P.O. Box 151, 84101 Beer-Sheva, Israel; 2 Internal Medicine ward E, Soroka University Medical Center, Beer-Sheva, Israel; 3 Department of Clinical Biochemistry and Pharmacology, Ben-Gurion University of the Negev, Beer-Sheva, Israel; 4 Nursing Research Unit, Soroka University Medical Center, Beer-Sheva, Israel

**Keywords:** Inflammatory bowel disease, ASCA, ANCA, Southern Israel, Arab Bedouin

## Abstract

**Background:**

Serological markers used for diagnostic purposes and disease stratification in inflammatory bowel disease.

We aimed to investigate the frequency of ASCA and ANCA among Arab Bedouin IBD patients and its relationship to disease phenotype and course.

**Methods:**

From cohort of 68, 25 Crohn’s disease (CD) and 25 Ulcerative colitis (UC) patients were recruited (72%). ASCA IgG was determined by ELISA assay. Immunofluorescence analysis of ANCA was performed.

**Results:**

The IgG ASCA was detected in 13 (52%) of the CD patients and in three (12%) UC patients. The prevalence of ANCA among UC patients was positive with 76%, sub-grouped, atypical ANCA in 9 patients (36%), pANCA in six patients (24%) and cANCA in 4 patients (16%).

The detection of ASCA among CD patients was found not to be a reliable predictor of young age at diagnosis, gender, ileal involvement, anti-TNF treatment or surgery.

UC patients with positive ANCA were younger, mean age 40.2 ± 11.9 compared with 57.3 ± 21.2 (*p* = 0.03), and diagnosed at a younger age, 29.2 ± 11.8 compared with 43.5 ± 15.3 (*p* = 0.05).

**Conclusion:**

The frequency of ASCA among Bedouin CD patients and ANCA among UC patients was high, however ASCA was not found to have a predictive value for disease phenotype or course. Positive ANCA in UC patients was predictive for younger age and age at diagnosis.

## Background

Inflammatory bowel disease (IBD) is a chronic inflammatory disorder of the gastrointestinal tract with two major forms, Crohn’s disease (CD) and ulcerative colitis (UC).

The Bedouin Arab populations are Muslims, who live in three areas in Israel, southern Israel (Negev desert), middle Israel and the Galil. In Southern Israel there are about 240.000 Arab Bedouins [1].

Our data from a previous report showed that there is an increase of the prevalence of (IBD) among the Bedouin population which could be attributed to the urbanization and modernization process with far-reaching lifestyle changes [2].

Diagnosis of IBD is based on a combination of clinical, radiological, endoscopic and histological criteria. Anti-*Saccharomyces cerevisiae* antibodies (ASCA) are antibodies directed against oligomannosidic epitopes of *Saccharomyces cerevisiae*, a strain of brewer’s yeast [3].

Anti-neutrophil cytoplasmic antibodies (ANCA) are autoantibodies, the major ANCA antigen targets in inflammatory vasculitides is the proteinase-3, which are cytoplasmic granular with accentuation between nuclear lobes (cANCA), wherever in IBD the myeloperoxidase are the target, which are fine homogenous, diffuse rim-like staining of perinuclear cytoplasm (pANCA) [4–6].The atypical pattern of ANCA is described as fine rime-like staining with intranuclear foci or broad nonhomogenous rim-like staining of the nuclear periphery as a mixture of c-ANCA and p-ANCA [7, 8].

In Western countries, increased levels of ASCA were found in 50–80% of patients with CD, in 20–25% of unaffected first-degree CD relatives, and only in a minority of UC patients (5–15%) and healthy subjects (0–5%) [9–13].

Positive p-ANCA are more common among UC patients, with the frequency of 40–80% of the UC patients [14].

The role of ANCA and ASCA in IBD though still controversial could have a diagnostic role, particularly in unclear cases, a previous report found a correlation with disease behavior. ASCA and ANCA may predict the development of IBD years before it is clinically diagnosed at the early age of disease onset, fibrostenosis, internal fistulas, perianal disease and ileal involvement, ASCA positive CD patients had an increased risk for early surgery [13, 15–18].

### Objective

Our aim of the present study was to investigate the frequency of ASCA and ANCA as serologic markers of IBD among the Bedouin Arab IBD patients. A second aim was to assess the association between the presence of ASCA or ANCA and clinical features of the disease.

## Methods

Patients: Arab Bedouin patients with known CD or UC were included in the present study. Fifty patients of 68 Bedouin Arab IBD patients in southern Israel were available for collection of the blood sample.

Demographic and clinical data for all included participants were reviewed carefully. The Data on demographics, the extent of disease, medical therapy, surgery, disease classification, complications, smoking and family history of IBD were obtained via questionnaire and review of patient’s records.

For CD and UC, the Montreal classification was used as previous described [19].

The study was carried out in accordance with the principles of the Helsinki Declaration. The study protocol was approved by the Institutional Ethics Committee, Helsinki committee at the Soroka University Medical Center, the approval number is 0036–14.

### ASCA

Patient serum samples were analyzed by a standardized enzyme-linked immunosorbent assay (ELISA) using antibodies ASCA IgG and also obtained by using INOVA QUANTA LiteTM ASCA kit [20].

The reactivity for each sample were calculated by dividing the average absorbance of the sample by the average absorbance of the ASCA IgG ELISA Low Positive and is multiplied by the number of units assigned to the ASCA IgG ELISA Low Positive.

Results of 0–20 was considered as negative, and 20.1–24.9 as equivocal and ≥ 25 as positive [20].

The sensitivity of the assay was reported previous as 74.4% for CD and 14.2% for UC [20].

### ANCA

ANCA testing was performed using Immunofluorescence analysis of pANCA, cANCA and atypic ANCA by a kit from Inova Diagnostic inc, San Diego, CA USA. The positive results were therefor analyzed for anti- Myeloperoxidase (MPO) antibodies and anti proteinase3 (PR3) [21].

Negative Reaction of ANCA was considered negative if specific nuclear and cytoplasmic staining is equal to or less system Negative Control. Positive sample was considered if specific nuclear and/or cytoplasmic staining was observed to be greater than the negative control [21].

### Statistical analysis

The results are presented as the mean (± SD) for continuous variables and the percentage of total patients for categorical data. The statistical analysis was carried out using SPSS IBM, version 21. For the categorical variables, proportions were compared using a t-test or χ2, as appropriate.

Significant differences in ASCA titers for different CD phenotypes were assessed using the Mann-Whitney U test. A *p*-value less than or equal to 0.05 was considered statistically significant.

## Results

A total of 50 IBD Bedouin patients, 25 CD and 25 UC were enrolled in our study, which accounts for 73% of the entire cohort of Bedouin Arab IBD patients in southern Israel.

### ASCA and ANCA frequency

The ASCA and ANCA frequency in CD and UC patient are summarized in Table 1. ASCA is more common among CD patients, with 52% of the CD patients compared with 12% in UC patients (*p* = 0.002). Among the 25 included UC patients, the most common type of ANCA was atypical ANCA, 36% (9/25).

**Table 1:** The frequency of ASCA and ANCA among CD and UC patients

	CD *n* = 25 (%)	UC *n* = 25 (%)
ASCA-positive	13 (52)	3 (12)
ANCA-positive		
pANCA	2 (8)	6 (24)
cANCA	2 (8)	4 (16)
atypical ANCA	1 (4)	9 (36)

### ASCA and CD

No significantly differences were found regarding age and age at diagnosis among ASCA positive and negative CD patients. None of the other clinical parameters including localization of the disease, surgery or treatment with Anti-tumor necrosis factor (anti-TNF) was significantly different among the ASCA positive patients compared with the ASCA negative CD patients. The clinical characteristics of the CD patients are summarized in Table 2.

**Table 2:** Clinical Characteristics of ASCA-positive and ASCA-negative CD disease cohort

Characteristic	ASCA positive *n* = 13 (52%)	ASCA negative *n* = 12 (48%)	*p*-value
Age	35.0 ± 9.1	38.1 ± 11.6	0.65
Age at diagnosis	26.2 ± 6.9	27.6 ± 9.8	0.89
Gender-male	8 (61.5)	7 (58.3)	0.87
Appendectomy	3 (25)	2 (18.2)	0.69
Family history of IBD	2 (15.4)	3 (25)	0.54
Smoking	5 (38.5)	3 (25)	0.47
Anti TNF treatment	6 (50)	4 (33.3)	0.43
Passed surgery	4 (30.8)	6 (50)	0.32
Disease localization			
Ileal	5 (38)	4 (33.3)	0.61
L2	0 (0)	2 (16.7)	0.61
L3	6 (46)	6 (50)	0.61
L4	2 (15.4)	0 (0)	0.61

### ANCA and UC

For the UC group, all three types of ANCA were found; positive c-ANCA was detected in 16% (4/25), p-ANCA in 24% (6/25) and atypical ANCA in 36% (9/25).

Of the patients with UC with negative ANCA or cANCA 90% (9/10) were male, while among patients with positive atypical ANCA or positive pANCA only 20% (3/15) were male (*p* = 0.002).

In the comparison between patients who tested positive for ANCA with those who tested negative for ANCA, we found out that the patients with positive ANCA were younger, 40.2 ± 11.9 years compared with 57.3 ± 21.2 years (*p* = 0.03) and the age at diagnosis was 29.2 ± 11.8 years compared 43.5 ± 15.3 years (*p* = 0.05). There was no relationship regarding other clinical parameters that was found. The clinical characteristics of the UC patients are summarized in Table 3.

**Table 3:** Clinical Characteristics of ANCA-positive UC patients

Characteristic	cANCA *n* = 4 (16%)	pANCA *n* = 6 (24%)	atypicANCA *n* = 9 (36%)	*p*-value
Age	44.0 ± 17.6	35.5 ± 14.2	41.6 ± 7.4	0.52
Age at diagnosis	23.0 ± 13.6	26.8 ± 14.1	33.5 ± 8.6	0.29
Gender-male	4 (100)	1 (16.7)	2 (22.2)	0.01
Appendectomy	0 (0)	0 (0)	0 (0)	–
Family history	0 (0)	1 (16.7)	0 (0)	0.32
Smoking	1 (25.0)	1 (16.7)	0 (0)	0.33
Passed surgery	1 (25.0)	0 (0)	1 (11.1)	0.49
Localization				
Proctitis	0 (0)	1 (16.7)	2 (22.2)	0.38
left Colitis	1 (25.0)	4 (66.7)	3 (33.3)	0.38
Pancolitis	3 (75.0)	1 (16.7)	4 (44.4)	0.38

## Discussion

IBD prevalence in Bedouin Arab population is increasing [2]. ASCA and ANCA are very well-studied markers in CD and UC as diagnostic markers or as a predictor for disease phenotype. No data were reported regarding these serological markers in the Arab Bedouin population.

We investigated the frequency of these markers and its association with the disease phenotype. This is the first report of IBD serologic markers Arab Bedouin IBD patients in southern Israel.

ASCA are known to be predominantly associated with CD and ANCA with UC.

In our study we found the frequency of 52% of ASCA in CD patients. Among the UC patients, we also found a frequency of 36% of atypical ANCA, whereas the frequency of pANCA was 24% of the UC cohort.

The frequency range of ASCA and ANCA are so wide and different in literature and in different population. The frequency of ASCA in CD patients and ANCA in UC patients may reach 80% in some reports [9–14].

In this study, we found a high frequency of ASCA and pANCA. However, in our cohort the most frequent ANCA type was the atypical ANCA and not the pANCA.

There are only few reports in the Arab world regarding serological markers in IBD among the Arab population; a study of IBD in children from Saudi Arabia found a prevalence of 35% of ASCA in CD patients and 28% of pANCA in UC [22].

Bread and beer are important sources of *Saccharomyces cerevisiae* yeast, only scant paper published regarding the relationship between the diet and ASCA frequency. However, no support of relationship between bread consumption and ASCA frequency was found in the scientific literature [23].

An important part of the nutrition among the BA is bread, which is included in the daily nutrition [24], and a low percentage of alcohol, we found a relative high frequency of ASCA among CD patients, the possible relationship between high consumption of bread and frequency of ASCA among this population is still to investigate in the future.

On the basis of the results of the present study, no relationship was found between the ASCA positivity and the disease phenotype characteristics in the CD patient’s cohort.

Also, in previous literature there are a large number of reports regarding the relationship between ASCA and CD phenotype; however, the results are controversial and vary among the previously reported studies, some of them found an association between ASCA positivity and early disease onset, longer disease duration, ileal involvement, complicated disease and IBD-related surgery [13–18]. Other several independent studies found no association between these parameters and ASCA positivity [25], it is therefore, important to mention is that these studies were heterogeneous in term of design, inclusion criteria, number of patients and other parameters of methodology [25]. In our work, the patients with positive ASCA were younger; however, it’s still none statistically significant. In our cohort we didn’t find a relationship between ASCA positivity and gender, family history of IBD, smoking, disease location, anti-TNF treatment or surgery.

While it is possible that there is no relationship between ASCA and disease phenotype in our population, from another view the no relationship perhaps could be linked to the small number of IBD patients in this specific population. It has also been suggested that to investigate this issue in this specific population in the future, when the number of IBD patients is larger, which could make the relationship clearer.

According to our results, the most common type of ANCA in Arab Bedouin IBD patients is the atypical ANCA, which was common by 36%. Several previous reports indicated a high frequency of atypical ANCA in other populations [26, 27]. In the present research the frequency of pANCA was 24%. This, therefore, translate into 60% of our UC cohort tested positive for pANCA or Atypical ANCA. In comparing our results with those in previous literature particularly Arab children in Saudi children, which showed a 28% frequency of pANCA [22], the frequency of pANCA and atypical ANCA is high in our cohort. To the best of our knowledge there are no other reports from Arab population in the Arab World.

In contrast to the more accurate results seen in our UC cohort**,** we have demonstrated that male patients are more likely to have negative ANCA or cANCA rather than atypical or pANCA. Of the ten patients who had negative ANCA or positive cANCA, nine of them were male (90%), however only 3 of 15 patients (20%) with positive pANCA or atypical ANCA were male (*p* = 0.002).

Atypical ANCA testing might be a helpful test for differentiating UC from CD in this specific population. Previous study suggested that atypical p-ANCA is a useful parameter to differentiate UC from CD [26].

In one hand, we have reported before, that the IBD among Bedouin Arab is increasing [2], which is attributed to change of lifestyle, including urbanization and modernization with a western lifestyle, particularly change of hygiene and nutrition. In the other hand we found a high prevalence of ASCA and ANCA positivity in the specific population. The Bedouin Arab in southern Israel are a very young society, 60% of the population are younger than age 19 [1]. The compensation of these factors let us expecting continuation increase of IBD incidence and prevalence in the future. The importance of this study might be the detecting a high frequency of serologic markers and its implication as diagnostic markers or as marker, which could differentiate between CD and UC.

The present study is limited by the small number of patients, the use of only the ASCA IgG type and the lack of control group.

## Conclusion

We found a high prevalence of ASCA in CD patients among Arab Bedouin IBD patients without relationship to the disease phenotype. A high frequency of atypical ANCA and pANCA were found among the UC patients with a prediction for younger age at diagnosis.

## Abbreviations

ANCA: Anti-neutrophil cytoplasmic antibodies
ASCA: Anti-*Saccharomyces cerevisiae* antibodies
CD: Crohn’s disease
IBD: Inflammatory bowel disease
UC: Ulcerative colitis
